# Psychotic Experiences and Hikikomori in a Nationally Representative Sample of Adult Community Residents in Japan: A Cross-Sectional Study

**DOI:** 10.3389/fpsyt.2020.602678

**Published:** 2021-01-29

**Authors:** Naonori Yasuma, Kazuhiro Watanabe, Daisuke Nishi, Hanako Ishikawa, Hisateru Tachimori, Tadashi Takeshima, Maki Umeda, Norito Kawakami

**Affiliations:** ^1^Department of Mental Health, Graduate School of Medicine, The University of Tokyo, Tokyo, Japan; ^2^Department of Community Mental Health and Law, National Institute of Mental Health, National Center of Neurology and Psychiatry, Kodaira, Japan; ^3^Department of Clinical Epidemiology, Translational Medical Center, National Center of Neurology and Psychiatry, Kodaira, Japan; ^4^Kawasaki City Center for Mental Health and Welfare, Kawasaki, Japan; ^5^Research Institute of Nursing Care for People and Community, University of Hyogo, Akashi, Japan

**Keywords:** hikikomori, social withdrawal, psychotic experiences, hallucinatory experiences, delusional experiences

## Abstract

Psychotic experiences (PEs) may be associated with hikikomori. In the present study, we analyzed interview data from a community-based representative sample (*N* = 1,616) in Japan to know the association of PEs over a life time, as well as the two components, hallucinatory experiences (HEs) and delusional experiences (DEs), with lifetime experience of hikikomori (severe social withdrawal). Logistic regression analysis was used to estimate the association between any PE, any HE, and any DE; and hikikomori, adjusting for socio-demographics and other psychopathologies (mental disorder in the past 12 months or having autistic spectrum disorder trait). Any PE was significantly associated with hikikomori [odds ratio (OR) =3.44, 95% CI = 1.14–10.33] after adjustment for sociodemographic factors, although the association attenuated after adjusting for other psychopathologies. Any DE remained significantly associated with hikikomori, even after adjustment for all the covariates (OR = 10.50, 95% CI = 1.57–70.29). Any HE was not significantly associated with hikikomori. DEs may be associated with hikikomori. However, because the study sample was small and the temporal association between DEs and hikikomori was unclear, a future study is needed to examine a causal relationship between DEs and hikikomori.

## Introduction

Hikikomori (severe social withdrawal) is a public mental health concern worldwide, as well as in Japan (1). Hikikomori is defined as withdrawing from all social engagement (e.g., education, employment, and friendships) for at least 6 months (2). The epidemiological research has shown the prevalence of hikikomori to be ~1% in Japan (3). Considerable concern has been growing about the potential societal and economic impact of hikikomori, such as a declining labor force, although the actual impact has yet to be examined (4–6). Meanwhile, it is important to understand its determinants and to develop preventive measures.

Previous studies investigated a variety of factors associated with the occurrence of hikikomori, including socio-cultural influences, e.g., the social structure, the school context, and family relationships (7–9). In addition, they identified a broad range of psychopathology, such as common mental disorders (3), Internet addiction (10), social phobic tendencies (11), and autistic spectrum disorders (12). Another previous study also reported a high prevalence of severe mental disorders among patients with hikikomori who sought help at community mental health centers: for instance, 5.3% of the cases were diagnosed as schizophrenia and other psychotic disorders (13). However, the sample may be biased because this previous study used only service users; the magnitude of the association between the disorder and hikikomori was unclear because no comparison was made with non-cases.

Psychotic experiences (PEs) refer to a broad spectrum of hallucinatory and delusional symptoms from clinical to subthreshold, that often are milder than symptoms of schizophrenia (14, 15). PEs are common in the general population: a previous meta-analysis reported a lifetime prevalence of 7.2% (16). This prevalence is much higher than that of schizophrenia (17). PEs have been recognized as a risk factor for developing psychotic disorders (18, 19), common mental disorders (20), and suicidality (21–23). Thus, PEs may be an important psychopathology that could affect various aspects of mental health and mental disorders. PEs also may be associated with the development of hikikomori. Previous studies revealed that PEs were associated with later poorer social functioning, and not participating in education, employment, or training (NEET) (24–26). Another previous study reported a high prevalence of schizophrenia, a severe form of PE, among cases with hikikomori (13). However, no previous study has investigated the association between PEs and hikikomori. Because PEs are milder psychotic symptoms common in the general population, studying the association between PEs and hikikomori would contribute to better understanding the psychopathology of hikikomori and developing effective early intervention measures.

PEs have two components: hallucinatory experiences (HEs) and delusional experiences (DEs) (27). These two components differ in terms of their prevalence, demographic characteristics, and social functioning (27–30). For example, a lifetime prevalence was found to be much greater for HEs (5.2%) than for DEs (1.3%) (27). HEs are more common among women, but no gender difference was found for DEs; DEs are more common in the younger generation, but no age difference was observed for HEs (27). A metacognitive decline was more prominently associated with DEs than with HEs (28–30). These previous findings suggest that HEs and DEs each have unique etiology and clinical consequences. It would be interesting to investigate the association with hikikomori separately for HEs and DEs, which also would contribute to understanding the psychopathology of hikikomori.

This cross-sectional study aimed to preliminarily explore the association between lifetime experienes of PEs and hikikomori in a national sample of community-living adults in Japan, based on the World Mental Health Japan Second Survey [WMHJ2; (31)]. While the study could not specify the causal relationship between PEs and hikikomori, it would provide a useful insight into this research topic that was poorly studied previously.

## Methods

### Sample

We conducted the WMHJ2 Survey by using a two-stage random sampling method from 2013 to 2015 among community residents in Japan (31). The survey area was divided into 3 sections: (1) Kanto district (Tokyo Metropolitan area and surrounding areas); (2) Tokai, Hokuriku, Koshin'etsu, Tohoku, and Hokkaido (the eastern part of Japan except for Kanto); and (3) Kinki, Chugoku, Kyushu, Shikoku, and Okinawa (the western part of Japan). We recruited a sample of almost 5,000 representative individuals aged 20–75 years old in 129 municipalities. Of these, 2,450 individuals participated in the studies and the average response rate was 43.4%. A face-to-face interview survey and a self-administered questionnaire were carried out by trained interviewers who visited the homes of respondents and collected the data. The Japanese computerized edition of the World Health Organization Integrated International Diagnostic Interview (WHO-CIDI), version 3.0 was used for the interview survey (32, 33). The Research Ethics Committee of the Graduate School of Medicine, The University of Tokyo approved the protocols of this study [nos. 10131-(1), -(2), -(3), and -(4)].

### Measures

#### Hikikomori

Hikikomori was measured by a single item, defined as a state of social withdrawal for more than 6 months, communicating only with family members and rarely leaving the house for work or school, measured by a module developed in a previous study. Participants were classified into those with or without any experience in the lifetime (yes/no; 3). Respondents were asked if they had ever experienced hikikomori [e.g., “Have you ever had a time when you stayed at home for more than 6 months without going to work or school or interacting with people other than your family (you may sometimes go shopping)?”].

#### Psychotic Experiences (PEs), any PE, any HE, any DE Over a Lifetime

The CIDI Psychosis Module was composed of six PE types. The six PE types consisted of two items related to HEs (visual hallucinations, auditory hallucinations) and four items related to DEs (thought insertion/withdrawal, mind control/passivity, ideas of reference, plot to harm/follow) (27, 32). Respondents were asked questions concerning PEs (see Appendix 1). Then, a probe question was asked to determine whether the reported PEs occurred when the patient was “not dreaming, not half asleep, and not under the influence of alcohol or drugs.” Only the latter type of responses were considered. We showed the prevalence estimates for any PE, any HE (with or without an associated DE) and any DE (with or without an associated HE) in this study.

#### Mental Disorder in the Past 12 Months

Mental disorder in the past 12 months was measured by using the WHO-CIDI 3.0 (31, 33). Mental disorders included 12 common mental disorders [generalized anxiety disorder, panic disorder, social phobia, agoraphobia, post-traumatic stress disorder, major depressive disorder, bipolar disorders (I and II), dysthymia, alcohol abuse and dependence, and drug abuse and dependence] which were diagnosed by using the Diagnostic and Statistical Manual of Mental Disorders, 4th edition (DSM-IV). Those reporting a common mental disorder in the past 12 months met the diagnostic criteria for any of these mental disorders. We measured autistic spectrum disorder (ASD) trait using the Japanese version of Autism Spectrum Quotient Short Form [AQ-J-10; (12, 34)]. AQ-J-10 is a 10-item self-administered questionnaire to screen adolescents and adults for current high-functioning pervasive developmental disorders. A total score of 7 or higher was defined as “having ASD trait” (34). The sensitivity and specificity of this cut-off point were 0.75 and 0.90, respectively (34).

#### Sociodemographic Factors

Age, gender, education, and income were examined as sociodemographic factors. Education was divided into four groups: junior high school graduates, high school graduates, some college, and university graduates or higher. Annual household income included the respondent's own earned income, spouse's income, income from others, social security income, and other income. We imputed the missing values of income by their age, sex, education, employment status and household size (35). However, we used those without missing values for other variables. Annual household income was divided into 4 groups: < 2.5 million yen, from 2.5 million yen to < 5 million yen, from 5 million yen to < 7.5 million yen, and 7.5 million yen or over.

### Statistical Analysis

Logistic regression analysis was used to estimate the association between any PE and hikikomori, any HE and hikikomori, and any DE and hikikomori. Hikikomori was analyzed as a dependent variable. Sociodemographic variables were adjusted for in Model 1 and Mental disorder in the past 12 months and having ASD trait were additionally adjusted for in Model 2. Fisher's exact test was performed to examine the association between the proportions of PEs and the presence of hikikomori. SPSS (Windows version 26) was used for statistical analysis and a *p* < 0.05 was considered statistically significant.

## Results

### Demographic Characteristics and Prevalence of Hikikomori and PEs

Among 2,450 total survey respondents, 1,775 answered the questionnaire on hikikomori. Some of these respondents had missing values on demographic variables (*n* = 6), PEs (*n* = 27), and ASD trait (*n* = 126); these respondents were excluded, which left an analytic sample of 1,616 respondents. Table 1 shows the demographic and psychosocial characteristics and the prevalence of hikikomori and PEs. For hikikomori, the average age in the survey was 37.38 (SD = 1.90), the average onset age was 27.53 (SD = 10.22), and the average hikikomori duration was 5.85 years (SD = 4.47). For PEs, the average age in the survey was 42.59 (SD = 1.50), and the average onset age was 24.21 (SD = 14.14). Thirty-four (2.1%) had experienced hikikomori and 1,582 had not. Among those who had experienced hikikomori, 4 had any PE, 2 had any HE, and 2 had any DE. Proportions with any PE (*p* = 0.04) and any DE (*p* = 0.01) among those who had experienced hikikomori were significantly higher than among those who had not experienced it, but the proportion with any HE was insignificant (*p* = 0.33). These *p*-values were obtained by Fisher's exact test.

**Table 1:** Demographics and psychosocial characteristics of the participants (*N* = 1,616).

	***N* (%), Mean (SD)**
Age (mean)	Mean = 44.16 (SD = 12.34)
Sex (men)	760 (47.0)
Education	
Junior high school	66 (4.1)
High school	580 (35.9)
Some college	474 (29.3)
University or higher	496 (30.7)
Household Income (per year)	
≤2.5 million yen	446 (27.6)
≤5 million yen	429 (26.5)
≤7.5 million yen	336 (20.8)
>7.5 million yen	405 (25.0)
Hikikomori	34 (2.1)
Any psychotic experience (PE) (over the lifetime)	63 (3.9)
Any hallucinatory experience (HE) (over the lifetime)	56 (3.5)
Any delusional experience (DE) (over the lifetime)	9 (0.6)
Mental disorder in the past 12 months (yes)	102 (6.3)
Having autistic spectrum disorders (ASD) trait (yes)	82 (5.1)

*Any PE, Any HE, Any DE, and Mental disorder in the past 12 months were diagnosed by using the Japanese computerized edition of the World Health Organization Integrated International Diagnostic Interview (WHO-CIDI), version 3.0. Having ASD trait was assessed by the Japanese version of Autism Spectrum Quotient Short Form (AQ-J-10)*.

### Any PE, any HE, any DE, and Hikikomori

As is seen in Table 2, any PE was significantly and positively associated with hikikomori after adjusting for socio-demographic variables (Model 1: OR = 3.44, 95% CI = 1.14–10.33). However, no significant associations were seen after adjusting for mental disorder in the past 12 months and ASD trait (Model 2: OR = 2.56, 95% CI = 0.80–8.34). In the sensitivity analyses, any HE was not significantly associated with hikikomori after adjusting for both Model 1 and Model 2 (OR = 1.83, 95% CI = 0.42–7.95; OR = 1.37, 95% CI = 0.29–6.45). On the other hand, any DE was significantly and positively associated with hikikomori after adjusting for both Model 1 and Model 2 (OR = 14.64, 95% CI = 2.50–85.60; OR = 10.50, 95% CI = 1.57–70.29).

**Table 2:** Psychotic experiences and Hikikomori: Logistic regression analysis (*N* = 1,616).

	**Hikikomori**					
	**Model 1**			**Model 2**		
	**OR**	**95%CI**	***p***	**OR**	**95%CI**	***p***
Any PE (over a lifetime)	3.44	1.14–10.33	0.03^*^	2.56	0.80–8.34	0.11
Any HE (over a lifetime)	1.83	0.42–7.95	0.42	1.37	0.29–6.45	0.69
Any DE (over a lifetime)	14.64	2.50–85.60	<0.01^**^	10.50	1.57–70.29	0.02^*^

*Odds Ratio (OR) and 95% confidence intervals (95%CIs) are shown. Sociodemographic variables were adjusted for in the Model 1; Mental disorder in the past 12 months, having ASD trait were additionally adjusted for in the Model 2*.

* *P < 0.05*,

** *P < 0.01*.

## Discussion

### Main Findings

To our knowledge, this is the first study to describe the association between PEs and hikikomori. Any PE and any DE were significantly and positively associated with hikikomori, while any HE was not significantly associated with hikikomori. Only any DE had a significant relation with hikikomori when adjusting for both sociodemographics and psychopathology. However, because this study was cross-sectional, we could not specify the temporal association between these two conditions. It was unclear if PE led to hikikomori, hikikomori led to PE, or PE and hikikomori co-occurred. In addition, because of the small sample size and low prevalence of PEs and hikikomori, the statistical power of the study, and the generalization of the findings may be limited.

### Comparing to the Previous Findings

Previous studies revealed that PEs were associated with later poorer social functioning and participation (24, 25). In addition, a previous survey revealed that hikikomori showed a wide coexistence with psychiatric disorders, including schizophrenia, mood disorders, anxiety disorders, personality disorders, and pervasive developmental disorder (36). Our results were consistent with the previous studies, and also found that any PE, particularly any DE, could also be one of the determinants for hikikomori.

### Potential Mechanisms

The possible mechanisms for the relationship between PEs and hikikomori is that PEs could be a determinant for hikikomori. Hikikomori was often observed in prodromal-stage cases of psychosis (37). Hallucinations and delusions might cause frightening experiences when outside and lead to hikikomori.

The reason that any DE, but not any HE, was significantly associated with hikikomori may be explained by the hypothesis that a DE could have mediated metacognitive decline and hikikomori. A previous study indicated that metacognitive decline could be associated with hikikomori (38). In addition, the metacognitive decline was reported to be less associated with hallucinations but more related to delusions (28–30). Therefore, it is possible that hikikomori could be caused by metacognitive decline mediated by delusion. That is why only any DE had a significant association with hikikomori.

### Strengths and Limitations

The strengths of this study are as follows. First, this study used a nationally representative sample using a two-stage random sampling method. Second, the study measured PEs, hikikomori and mental disorders diagnosed by a structured interview, WHO-CIDI 3.0.

However, there are many limitations in this study. Thus, caution should be used in interpretation of the results. First, as we stated earlier, this study was cross-sectional, which could not show a causality between PEs and hikikomori. We could not specify the temporal association between these two conditions because data were limited in this study. The alternative explanation was that hikikomori could affect PEs. Sensory deprivation has been linked to psychotic symptoms for decades, even in otherwise normal individuals (37). The sensory deprivation experienced by hikikomori who stay for extended periods in their room could increase PEs. Another explanation was the social defeat (SD) hypothesis, which is that long-term exposure to the experience of SD may lead to sensitization of the mesolimbic dopamine system and thereby increase the risk for schizophrenia (39, 40). People with hikikomori could have felt SD, which would increase the potential for PEs. However, to clarify the potential mechanism, longitudinal studies should be conducted in the future. Second, the number of cases with PEs and hikikomori was small as a proportion of our overall sample size. The sample may include selected participants that limit the generalizability. We may have overlooked some important associations. Third, the response rate of the WMHJ2 survey was not high overall, which could cause selection bias, although a previous study of the prevalence of common mental disorders suggested that response rate might not have a strong effect on the prevalence estimates of mental disorders in a community-based survey (41). Fourth, information bias may have been present if respondents with PEs were more aware of problems related to hikikomori and therefore more hesitant to report such problems in the survey. Fifth, other covariates may confound the association; for instance, psychotic disorders such as schizophrenia, which was not measured in this study, may partly explain the association between PEs and hikikomori. We did not measure negative symptoms that are often observed among people with chronic schizophrenia, which could also be an important psychopathology leading to social withdrawal. Sixth, our definition of “hikikomori” following that in a previous study (42) was based on only the behavioral patterns and did not consider the etiology or psychopathology of hikikomori. Hikikomori may consist of multiple psychopathologies. The association between subtypes of hikikomori and PE needs to be investigated in future research.

### Implications

While the findings are largely limited by the small number of hikikomori cases and PE cases, some clinical implications can be drawn. Some Hikikomori cases may develop due to or at least be comorbid with PEs. Thus, psychiatric assessment of these cases would be important. By sharing with health professionals the finding that PEs could be a determinant of hikikomori, early intervention for PEs with careful assessment, frequent monitoring and, if necessary, outreach visits to homes could be a good practice. In addition, it could be useful if health professionals would provide sufficient information to the public about the known risks of PEs and enhance the consultation support system for people with PEs and their families. Carrying out these efforts may decrease the future risk for hikikomori in the population.

### Conclusions

Any PE, particularly any DE, might be associated with hikikomori in this general population sample. The findings obtained in the current study inform our understanding of the relationship between PEs and hikikomori. Future population-based studies with longitudinal data are needed to better understand the etiology and prevention of hikikomori in the context of PEs.

## Data Availability Statement

The raw data supporting the conclusions of this article will be made available by the authors, without undue reservation.

## Ethics Statement

The studies involving human participants were reviewed and approved by The Research Ethics Committees of the Graduate School of Medicine/Faculty of Medicine, The University of Tokyo [nos. 10131-(1), -(2), -(3), and -(4)] approved the aims and procedures of this study before it began. The patients/participants provided their written informed consent to participate in this study.

## Author Contributions

NY was the principal investigator who conceptualized and designed the study, analyzed data, and drafted the article. KW, DN, HI, HT, TT, MU, and NK made a critical revision of the article and gave final approval for the version to be published. All authors contributed to the article and approved the submitted version.

## Conflict of Interest

The authors declare that the research was conducted in the absence of any commercial or financial relationships that could be construed as a potential conflict of interest.
