# Supplementary material for: Psychotic Experiences and Hikikomori in a Nationally Representative Sample of Adult Community Residents in Japan: A Cross-Sectional Study
Source: Front Psychiatry. 2021 Jan 29;11:602678. doi: 10.3389/fpsyt.2020.602678 (PMC7878546; doi:10.3389/fpsyt.2020.602678)
Supplement: Supplementary file 1 [file Data_Sheet_1.DOCX]

**Appendix 1 CIDI Psychosis Module included questions about six PE types**

| Type 1. (Saw a vision) | Did you ever see something that wasn’t really there that other people could not see? |
| --- | --- |
| Type 2. (Heard voices) | Did you ever hear things that other people said did not exist, like strange voices coming from inside your head talking to you or about you, or voices coming out of the air when there was no one around. |
| Type 3. (Thought insertion) | Did you ever believe that some mysterious force was inserting many different strange thoughts -- that were definitely not your own thoughts – directly into your head by means of x-rays or laser beams or other methods? |
| Type 4. (Mind control/passivity) | Did you ever feel that your mind had been taken over by strange forces with laser beams or other methods that were making you do things you did not choose to do. |
| Type 5. (Ideas of reference) | Did you ever believe that some strange force was trying to communicate directly with you by sending special signs or signals that you could understand but that no one else could understand. Sometimes this happens by special signs coming through the radio or television. |
| Type 6. (Plot to harm /follow) | Did you ever believe that there was an unjust plot going on to harm you or to have people follow you that your family and friends did not believe existed? |
